# Prevalence of posttraumatic arthritis following distal radius fractures in non-osteoporotic patients and the association with radiological measurements, clinician and patient-reported outcomes

**DOI:** 10.1007/s00402-018-3046-2

**Published:** 2018-10-13

**Authors:** C. M. Lameijer, H. J. ten Duis, D. Vroling, M. T. Hartlief, M. El Moumni, C. K. van der Sluis

**Affiliations:** 1 Department of Trauma Surgery, University Medical Center Groningen, University of Groningen, Postbox 30.001, Huispostcode BA51, 9700 RB Groningen, The Netherlands; 2 Rehabilitation Center ‘Revalidatie Friesland’, Leeuwarden, The Netherlands; 3 Department of Rehabilitation Medicine, University Medical Center Groningen, University of Groningen, Groningen, The Netherlands

**Keywords:** Wrist, Distal radius, Posttraumatic arthritis, Patient-reported outcome

## Abstract

**Introduction:**

Outcomes of non-osteoporotic patients who sustained a distal radius fracture (DRF) have not gained much attention in recent literature. The aims of this study were to determine the prevalence of posttraumatic arthritis (PA), to analyze associations of radiological measurements, clinician-reported and patient-reported outcomes (CROs and PROs) with PA and gain insight into employment changes after DRF in non-osteoporotic patients.

**Methods:**

Non-osteoporotic patients following a DRF were selected. Radiographs of both wrists were obtained at follow-up and the degree of PA was determined. Radiological measurements consisted of grading of PA, ulnar variance, radial length, radial inclination, dorsal tilt, distal radio-ulnar joint width, scapholunate dissociation, step-off and gap. Active range of motion and grip strength measurements were performed and all patients filled in four questionnaires to assess pain, upper extremity functioning, and health status (Disability of Arm, Shoulder and Hand; Patient Reported Wrist Evaluation; Michigan Hand Questionnaire; Short Form-36).

**Results:**

Seventy-three patients (32 women, 41 men) with a mean age of 33.5 (SD 9.2) years were included. Prevalence of PA was 32% at a median follow-up of 62.0 months. Patients with PA had statistically significant longer radial length (1.1 mm, 95% CI − 2.1; − 0.0, *p* = 0.045). Patients with PA had a statistically significant diminished flexion/extension arc of motion (12.0°, *p* = 0.008) and ulnar/radial deviation arc of motion (6.3°, *p* = 0.018). When corrected for dominance, all grip strength measurements were not statistically significantly different between patients with and without PA. Statistically significant poorer PROs in patients with PA were the MHQ subscales general functioning (65 versus 75, *p* = 0.018), esthetics (94 versus 100, *p* = 0.037), satisfaction (75 versus 92, *p* = 0.042) and total score of the MHQ (83 versus 91, *p* = 0.044), as well as the SF-36 subscale physical functioning (95 versus 100, *p* = 0.028). In regression analyses the DASH, PRWE function and PRWE total were statistically significantly associated with flexion/extension arc of motion. Seven patients (10%) changed or left their occupation because of the DRF.

**Conclusion:**

Non-osteoporotic patients had a considerably high prevalence of PA following DRFs, despite a relatively short follow-up time. Patients with longer radial length more often had PA. Irrespective of AO/OTA fracture type, patients with PA had diminished range of motion, but no altered grip strength measurements. Non-osteoporotic patients following DRFs perceived diminished general functioning and dissatisfaction, which was impacted by the diminished active range of motion. Pain or impaired general health status was not reported. The PRO MHQ might be a valuable evaluation tool in this patient group. Change of occupation following DRFs should receive attention in further research.

## Introduction

The development of posttraumatic arthritis (PA) following distal radius fractures (DRFs) has been described in populations with a wide range in age and follow-up time [1]. Clinical studies have supported the hypothesis that an increasing age is an important risk factor for the development of PA [2]. However, already in non-osteoporotic patients the prevalence of PA following DRFs has been described as high as 43–50% [3, 4]. Since DRFs in young non-osteoporotic patients usually result from high energy trauma, these injuries often have intra-articular involvement [5]. This can result in residual articular incongruence, which is usually described in step-offs and gaps [6–11]. Intercarpal ligamentous injuries, radiologically reflected in the distance between scaphoid and lunate (SL ligament injury) and distal radio-ulnar joint instability are also associated with DRFs [5, 12, 13]. Conflicting results have been reported in literature with regard to other radiological parameters and their association with the development of PA in heterogeneous cohorts [4–6, 11, 14]. PA following a DRF has been associated with diminished clinician reported outcomes (CROs), such as active range of motion and strength measurements, in populations with wide age ranges [4–6, 15, 16]. Also, an association between PA following DRF and poorer patient-reported outcomes (PROs), assessed by the Short Form Health Survey 36 (SF-36) in patients with an age range of 24–93 years has been reported [17]. Other studies did not find an association between PROs and PA [18–20]. Literature suggests that patients with pre-existing osteoporosis who sustained a DRF have better PROs than those without osteoporosis [21, 22]. In addition to pre-existing osteoporosis, age seems to be an independent factor influencing PROs following DRFs [2].

Few studies report on non-osteoporotic study populations following DRFs [23, 24]. As a consequence, limited information is available on the long-term outcomes of non-osteoporotic patients following a DRF. Although high prevalence of PA is reported in literature in non-osteoporotic patients after DRF, associations with CROs and PROs remain unclear. We hypothesized that PA following DRFs in non-osteoporotic patients may have greater impact because an active (working) life may pose higher demands on wrist function compared to older patients. Therefore, insight in the association between radiological measurements and PA and the association between PA and wrist function, activity performance, pain, satisfaction, quality of life in these young patients is mandatory. This knowledge could be used to direct rehabilitation treatment and to inform young patients on outcomes and influence on societal roles (e.g. occupation) they can expect in the long-term.

### Objectives

The aims of this study were to determine the prevalence of PA, and to analyze associations of radiological measurements, CROs and PROs with PA and gain insight in employment changes following a DRF in non-osteoporotic patients.

## Methods

**Fig. 1:**
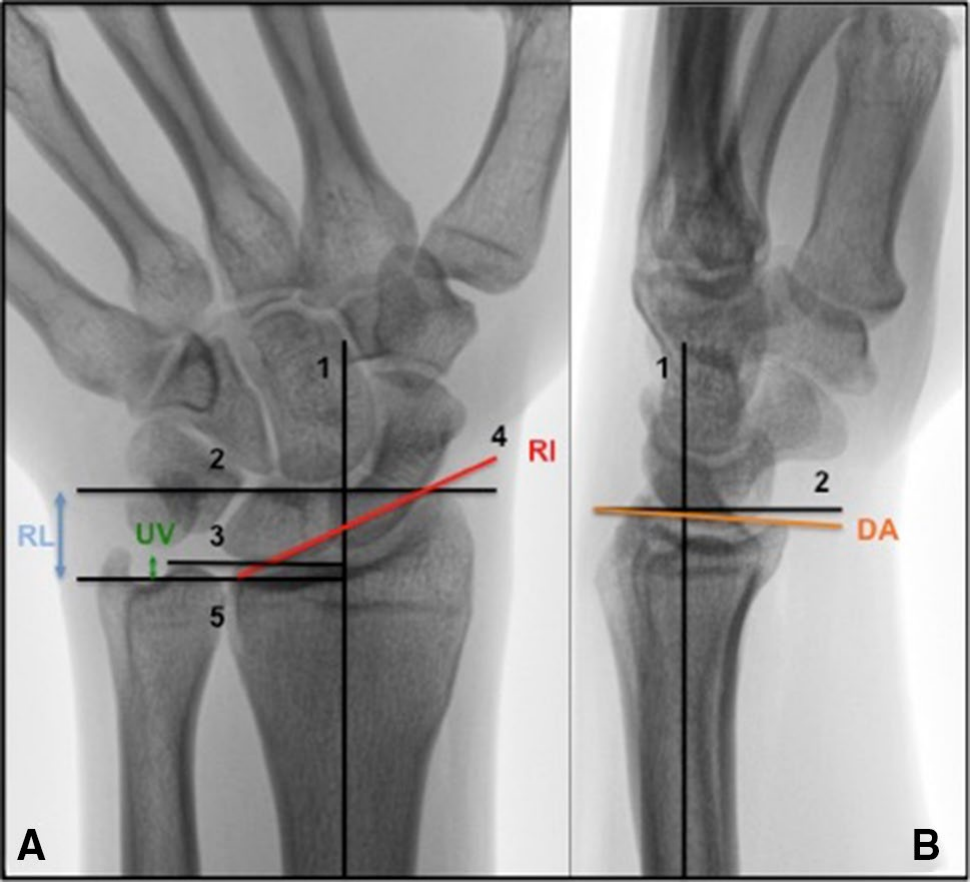
**a** Posteroanterior measurement guidelines: (1) The center of the radial shaft is determined at 3 cm and 5 cm below the mid-region of the proximal lunate articular surface. This line represents the central axis of the radius. (2) A line perpendicular to the central long axis of the radius is drawn at the level of the most distal aspect of the radial articular surface. (3) A line perpendicular to the central long axis of the radius is drawn at the level of the ulnar margin of the distal radial articular surface. (4) The radial and ulnar margins of the distal radial articular surface are connected. (5) A line perpendicular to the central long axis of the radius is drawn at the level of the distal ulnar articular surface. **b** Lateral measurement guidelines: (1) The center of the radial shaft is determined at 3 cm and 5 cm below the mid-region of the proximal lunate articular surface. This line represents the central long axis of the radius. (2) A line perpendicular to the central long axis of the radius is drawn at a convenient level. (3) The dorsal and anterior margins of the distal radial articular surface are connected. *UV* ulnar variation, *RL* radial length, *RI* radial inclination, *DT* dorsal tilt

**Fig. 2:**
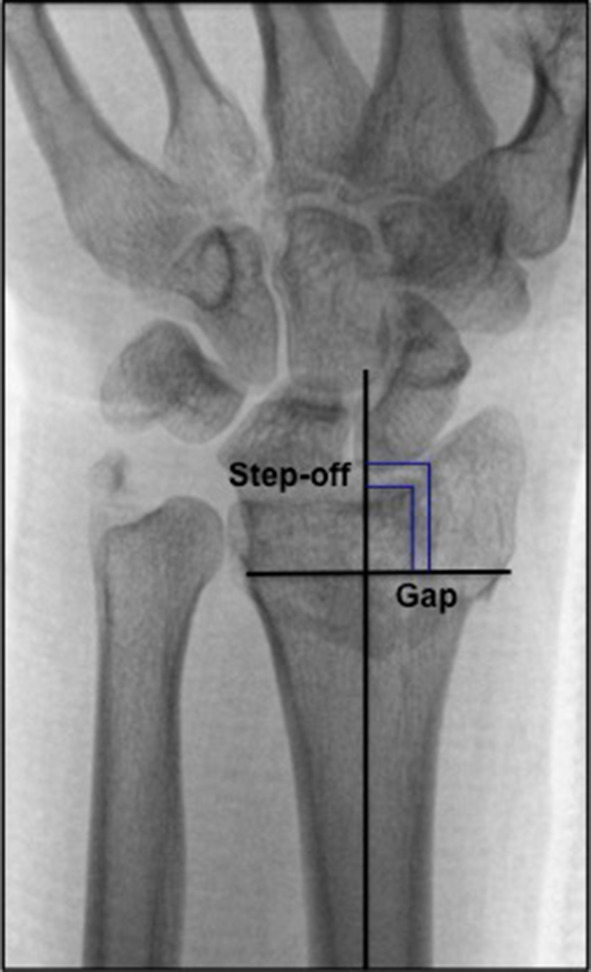
Step-off and gap measurement. (1) Step-off at the articular surface of the distal radius was measured parallel to the central long axis of the radius by drawing perpendicular lines from the most distal margin of each side of the articular incongruence. (2) Gap deformity was measured along a perpendicular line to the central long axis of the radius

This retrospective cohort study was approved by the Medical Ethics Committee (NL41587.099.13) and registered at the Dutch Trial Bureau (TC 4002). Before entering the study, participants signed an informed consent form. From a level II trauma center database, we selected all patients of a non-osteoporotic age group (men, 18–50 years and women, 18–40 years old, at the time of injury) who sustained a DRF between January 2005 and January 2011 and were treated both non-surgically or surgically [25–27]. Exclusion criteria were fractures treated surgically after the 7th day following injury, open fractures, pre-existing osteoarthritis or risk factors for early osteoporosis (steroid use, alcoholism or early menopause, low body weight), because outcomes in these patients might not be representative for young non-osteoporotic patients following a DRF. A total of 433 patients fulfilled the inclusion criteria and received an invitation to participate in the study. A notification of changed home address was received from 43 participants, but current addresses could not be retrieved. From 306 patients, no response was received. Eighty-four patients responded of which 73 (32 women, 41 men) consented to participate. All eligible patients were invited for a single visit to the rehabilitation department. One hand therapist measured CROs (active range of motion and grip strength). Patients also filled in four PROs at the time of their visit. At the time of the participants’ visit, lateral (Lundy) and posteroanterior (PA) wrist radiographs were made of both wrists. All radiographs were evaluated by a single radiologist specialized in musculoskeletal disorders with a special interest in hand and wrist anatomy. PA was classified according to the grading system as described by Knirk and Jupiter: 0 = no signs of PA, I = slight joint-space narrowing, II = marked joint-space narrowing and osteophyte formation, III = bone-on-bone, osteophyte and cyst formation [1]. Further radiological parameters were measured according to the technique described by Kreder et al.; ulnar variance, radial length, radial inclination and dorsal angulation (Fig. 1) and step-off and gap (Fig. 2) [28, 29]. In addition, the scapholunate distance (SL distance) and the distal radio-ulnar joint (DRUJ) space were measured [30]. Normal ranges for radiological factors have been previously described; ulnar variance − 4 to 2 mm [31, 32], radial length 8–17 mm [31], radial inclination 16°–29° [33, 34], dorsal angulation 0-palmar 22° [35, 36]. In addition, to correct for anatomical variation between patients, measurements of the uninjured wrist were obtained at follow up and used as a reference to interpret measurements of the injured wrist.

### CROs: active range of motion and grip strength

The participants were positioned sitting at a table, with hips and knees flexed 90°. Elbows were positioned on the table and flexed in 90° with wrists in neutral position. A digital protractor of Biometrics LTD and E-Link^Ⓡ^ software was used to measure active range of motion. Flexion/extension arc of motion, ulnar/radial deviation arc of motion and supination/pronation arc of motion were measured in degrees. Grip strength and sustained grip strength were measured in kilograms using a digital Jamar dynamometer and key pinch strength using a pinch meter of Biometrics LTD and E-Link^Ⓡ^ software. For people with right sided dominance it is known that the right hand has 10% more grip strength in comparison to the left hand. This is not the case when people are left sided dominant; grip strength in both hands is similar [37]. Therefore, a correction for grip strength measurements was performed to correct for right sided dominance. Grip strength of the injured wrist was calculated as a percentage of the uninjured wrist to correct for variation between patients. For assessing sustained grip strength, patients were asked to grip as hard as possible during a 30 s period, the average grip strength (kilograms), computed over the last 18 s of this 30-s period was recorded. Key pinch strength measured in kilograms was derived from the maximum peak strength sustained during at least 2 s. The mean of three performances was presented for all strength measurements. First, all active range of motion measurements were recorded. Subsequently grip strength, sustained grip strength and key pinch strength were assessed in consecutive order, alternating dominant and non-dominant sides.

### PROs: DASH, PRWE, MHQ, SF-36

All patients completed 4 questionnaires involving pain scores, specific upper extremity functioning, and health status.

The Disability of Arm, Shoulder and Hand (DASH) Questionnaire is a 30-item self-report measure assessing physical functioning and symptoms of the upper limb. DASH-scores range from 0 to 100 (higher scores indicate worse function). The DASH has a good validity, reliability and responsiveness in upper extremity disability assessment [38, 39].

The Patient Rated Outcome Evaluation (PRWE) is a 15-item questionnaire divided into two subscales: pain (5 items) and function (10 items). The PRWE was developed to assess pain and functioning in patients with DRFs [40]. The pain items were selected to represent the total spectrum of frequency and intensity. The function items were selected to represent a range of physical activities that require different ranges of motions or muscle strength capabilities. For both subscales the maximum score is 50 (most disability) and the minimum score is 0 (no disability). Although these subscales have been reported frequently in literature, it has been suggested that the PRWE measures a single dimensional trait, and a single (sum) score should be used [41]. The questionnaire has a good validity for symptoms and function of the wrist [42].

The Michigan Hand Outcomes Questionnaire (MHQ) assesses hand outcomes that are of importance to patients and specific for the impaired hand (left and right separately) and includes 6 subscales (general function, activities of general life, work, pain, esthetics and satisfaction). The subscale score is the sum of the outcome of each question and ranges from 0 to 100. A higher score on the pain subscale indicates less pain. For the other five subscales and the total score higher scores imply a better function. The MHQ compares favourably with other PROs regarding upper extremity in the area of test–retest reliability, validity and responsiveness. In addition it has high internal consistency [43]. The strength of the MHQ is its multidimensional construct in measuring symptoms, function, aesthetics and satisfaction [43].

The SF-36 is developed to survey overall health status [44]. It contains 36 questions to assess limitations in (1) physical function, (2) role function, (3) social function, (4) bodily pain, (5) general mental health, (6) limitations in role function due to emotional problems, (7) vitality and (8) general health perception. Scale scores range from 0 to 100 with higher scores indicating a better health status. Scale scores can be used to calculate a physical and a mental component summary score [44]. Validity of this questionnaire is sufficient for groups reporting varying extents of illness-health [45].

### Work

The intensity of executing work tasks was categorized according to the Dictionary of Occupational Titles (DOT) classification in sedentary, light, medium, heavy or very heavy work [46]. Patients filled in a short questionnaire to report change of work following the DRF and the reason for such a change.

### Statistics

Data were assessed for normal distribution. Continuous data were presented as mean (standard deviation, SD) and as median (interquartile range, IQR) when no normal distribution of the data was present. The Chi squared test and Fisher’s exact tests were used to analyze associations between dichotomous and/or categorical variables. *T* tests were performed when analyzing continuous variables if a normal distribution was found. If data did not have a normal distribution, Mann–Whitney *U* tests were used. One way ANOVA tests were performed when analyzing the association between continuous variables and categorical variables, Bonferroni posthoc analyses were performed afterwards. Multivariable linear regression analysis, using backward stepwise selection (until all *p* values were ≤ 0.2) was performed with PROs as an outcome and other factors (age, gender, AO/OTA fracture type, type of treatment, follow-up, flexion–extension arc of motion, grip strength and presence of PA) as explanatory variables. To be able to impute the categorical variable AO/OTA fracture type In regression analysis, 2 dummy variables were calculated with AO/OTA fracture type A as reference variable (dummy1 is AO/OTA fracture type B = 1, other types = 0; dummy2 is AO/OTA fracture type C = 1, other types = 0). Level of significance was set at *p* ≤ 0.05. All statistical analyses were performed using IBM SPSS, version 22.

## Results

Eighty-four patients of the 433 eligible patients responded to the invitation to participate in the study of which 73 (32 women, 41 men) consented to participate with a mean age of 33.5 (SD 9.2) years at the time of the injury (participation rate 19%) (Table 1). Participants suffered statisticaly significant more often from intra-articular fractures according to the AO foundation and Orthopaedic Trauma Association (AO/OTA) classification system than non-participants. Of the participants, 19.2% had type A fractures, 41.1% type B and 39.7% type C fractures. In contrast, of the non-participants 53.1% had type A fractures, 28.6% type B and 18.3% type C fractures (*p* = 0.013). No further differences between participants and non-participants were found.

**Table 1:** Patient characteristics of the total population and differences between patients with and without PA

	Total population (*N* = 73)	PA (*N* = 23)	No PA (*N* = 50)	Difference in means (95% CI)	*p* value
Age at time of the injury (years)					0.004*
Mean (SD)	33.5 (9.2)	38.0 (8.6)	31.4 (8.9)	6.6 (2.1; 10.9)	
Follow up (months)					0.771
Median (IQR)	62.0 (53.0; 84.5)	70.0 (56.0; 84.0)	62.0 (52.8; 85.0)		

	*N* (%)	*N* (%)	*N* (%)		
Gender					
Male	41 (56.2)	17 (73.9)	24 (48.0)		0.038*
Energy trauma					0.150
Low energy	20 (27.4)	3 (13.0)	17 (34.0)		
High energy	45 (61.6)	16 (69.6)	29 (58.0)		
Unknown	8 (11.0)	4 (17.4)	4 (8.0)		
AO/OTA classification					0.357
A	14 (19.2)	3 (13.0)	11 (22.0)		
B	30 (41.1)	8 (34.8)	22 (44.0)		
C	29 (39.7)	12 (52.2)	17 (34.0)		
Dominant hand injured	37 (50.7)	9 (39.1)	28 (56.0)		0.180
Treatment					0.100
Cast	33 (45.2)	7 (30.4)	26 (52.0)		
Closed reduction/cast	12 (16.4)	4 (17.4)	8 (16.0)		
Surgical	28 (38.4)	12 (52.2)	16 (32.0)		
Grading PA					
Gr 0	50 (68.5)	0	50		
Gr I	13 (17.8)	13	0		
Gr II	10 (13.7)	10	0		
Gr III	0 (0.0)	0	0		

Results of independent samples *T* test (age) and Chi-squared test (other variables)

*N* number of patients, *SD* standard deviation, *IQR* interquartile range, *PA* posttraumatic arthritis, *95% CI* 95% confidence interval, fracture type A, B and C according to the AO foundation and Orthopaedic Trauma Association AO/OTA classification, *PA* posttraumatic arthritis

*Statistical significance

### Prevalence of PA

After a median follow up of 5 years (62.0 months) the prevalence of PA (grade I, II) was 32% (Table 1). Patients with PA were more often males (73.9% versus 48.0%) and statistically significant older (6.6 years) than patients without PA (Table 1). No statistically significant differences between patients with and without PA regarding trauma energy, type of treatment, AO/OTA fracture classification or dominance were found (Table 1). Patients who were treated surgically, more often had AO/OTA type C fractures in comparison to patients who were treated conservatively (AO/OTA type A 1, type B 0, type C 27 surgically treated, AO/OTA type A 8, type B 18, type C 19 conservatively treated).

### PA and radiological measurements

All radiological measurements were within normal ranges [31–36]. Between patients who were treated surgically and patients who were treated conservatively (with our without closed reduction), no statistically significant difference in radiological measurements were found at follow up. When comparing the radiological measurements of the injured to the uninjured wrist at follow up, only dorsal angulation was statistically significant more pronounced in the injured wrist (− 1.3° versus 5.1°, *p* < 0.001) (Table 2). Patients with PA had a statistically significant longer radial length at follow up (13.7 mm versus 12.6 mm, *p* = 0.045) (Table 3). Also, the difference in radial length between the injured and uninjured wrist was greater in patients with PA (0.6 versus − 0.6, *p* = 0.024). All other radiological measurements at follow up did not differ between the patients with and without PA (Table 3).

**Table 2:** Radiological measurements at follow up compared with the measurements of the uninjured wrist at follow-up

Radiological factors	*N*	Follow up injured wrist		Follow-up uninjured wrist		Mean difference (SD)	Significance
		Mean	SD	Mean	SD		*p* (95% CI of mean difference)
Ulnar variance (mm)	73	0.9	1.8	0.4	1.6	0.4 (1.9)	0.063 (− 0.0; 0.9)
Radial length (mm)	73	13.0	2.1	13.2	2.1	− 0.2 (2.1)	0.318 (− 0.7; 0.2)
Radial inclination (°)	73	25.5	3.6	26.4	3.8	− 0.9 (4.2)	0.079 (− 1.9; − 0.1)
Dorsal angulation (°)	73	− 1.3	6.6	− 5.1	4.1	3.8 (6.5)	< 0.001 (2.3; 5.3)*
SL distance (mm)	45	2.1	0.4	2.0	0.4	0.1 (0.5)	0.099 (− 0.0; 0.3)
DRUJ distance (mm)	72	2.4	0.8	2.3	0.8	0.1 (0.7)	0.224 (− 0.1; 0.3)

Results of paired samples *T* test

*N* number of patients, *SD* standard deviation, *95% CI* 95% confidence interval, *SL* scapholunate ligament, *DRUJ* distal radioulnar Joint

*Significant difference

**Table 3:** Associations between radiological measurements and PA

Radiological factors	PA (*N* = 23)		No PA (*N* = 50)		Significance
	Mean	SD	Mean	SD	*p* (95% CI of mean difference)
Ulnar variance (mm)	1.1 (*N* = 23)	2.4	0.7 (*n* = 50)	1.5	0.462 (− 1.5; − 0.7)
Radial length (mm)	13.7 (*N* = 23)	2.5	12.6 (*n* = 50)	1.9	0.045* (− 2.1; − 0.0)
Radial inclination (°)	25.6 (*N* = 23)	4.4	25.5 (*n* = 50)	3.2	0.888 (− 1.9; 1.7)
Dorsal angulation (°)	− 2.2 (*N* = 23)	8.0	− 0.9 (*n* = 50)	5.9	0.472 (− 2.4; 5.2)
SL distance (mm)	2.3 (*N* = 16)	0.4	2.1 (*n* = 38)	0.4	0.072 (− 0.4; 0.0)

	Median	IQR	Median	IQR	Mann–Whitney *U* *p* value
DRUJ distance (mm)	2.1 (*N*23)	1.8; 2.9	2.3 (*n* = 50)	1.9; 2.8	0.533
Step-off (mm)	0.0 (*N* = 16)	0.0; 0.0	0.0 (*n* = 38)	0.0; 0.0	0.053
Gap (mm)	0.0 (*N* = 15)	0.0; 0.0	0.0 (*n* = 39)	0.0; 0.0	0.177

Outcome of Independent *T* test and Mann–Whitney *U*

*N* number of patients, *SD* standard deviation, *95% CI* 95% confidence interval, *SL* scapholunate ligament, *DRUJ* distal radioulnar joint

*Significant difference

### CROs

#### Active range of motion

Patients with PA had statistically significant diminished flexion/extension arc of motion (12°, *p* = 0.008) and ulnar/radial deviation arc of motion (6.3°, *p* = 0.018) compared to patients without PA (Table 4).

**Table 4:** CROs: differences between patients with and without PA

CROs	PA (*N* = 23) Mean (SD)	No PA (*N* = 50) Mean (SD)	Difference in means (95% CI)	*p* value
Active range of motion (°)				
Flexion/extension arc	133.1 (17.8)	145.1 (17.3)	12.0 (3.2; 20.7)	0.008*
Ulnar/radial deviation arc	53.7 (9.4)	60.1 (10.7)	6.3 (1.1; 11.5)	0.018*
Pro/supination arc	144.8 (14.2)	147.6 (12.4)	2.8 (− 3.7; 9.3)	0.397
Grip strength not corrected for dominance (% of the uninjured wrist)				
Grip strength	88.0 (15.7)	97.3 (17.4)	9.3 (0.8; 17.8)	0.032*
Sustained grip	90.3 (18.3)	98.9 (26.4)	8.6 (− 3.6; 20.8)	0.165
Key pinch strength	89.4 (18.0)	98.2 (11.6)	8.8 (0.4; 17.1)	0.015*
Grip strength corrected for dominance (% of the uninjured wrist)				
Grip strength	89.6 (11.8)	97.3 (16.7)	7.7 (− .1; 15.4)	0.052
Sustained grip	92.2 (17.9)	98.7 (28.5)	6.5 (− 6.3; 19.4)	0.315
Key pinch strength	115.5 (121.1)	108.7 (74.6)	− 6.8 (− 52.8; 39.2)	0.769

Results of independent samples *T* test

*N* number of patients, *SD* standard deviation, *PA* posttraumatic arthritis, *95% CI* 95% confidence interval

*Statistical significance

AO/OTA fracture classification was not statistically significant associated with active range of motion (Table 5).

**Table 5:** Associations between AO/OTA classification and active range of motion

AO/OTA classification	One way ANOVA Active range of motion (°)				Posthoc Bonferroni Difference in means (SE)					
	Type A Mean (SD)	Type B Mean (SD)	Type C Mean (SD)	*p*	A versus B	*p*	A versus C	*p*	B versus C	*p*
Flexion/extension arc	150.1 (17.0)	139.1 (19.2)	139.2 (16.9)	0.128	11.0 (5.8)	0.074	10.9 (5.8)	0.434	− 0.1 (4.7)	1.00
Ulnar/radial deviation arc	56.7 (9.8)	57.6 (12.2)	59.2 (9.7)	0.742	− 0.9 (3.5)	1.00	− 2.5 (3.5)	1.00	− 1.6 (2.8)	1.00
Pro/supination arc	150.0 (10.3)	146.3 (12.8)	145.5 (14.4)	0.563	3.7 (4.2)	1.00	4.5 (4.3)	0.887	0.8 (3.4)	1.00

Results of one way ANOVA and posthoc Bonferroni tests

*SD* standard deviation, *SE* standard error

#### Grip strength

Compared to patients without PA, the grip strength and key pinch strength were 9.3% and respectively 8.8% weaker (*p* = 0.032 and *p* = 0.015) in patients with PA (Table 4). However, when correcting for dominance with the 10% rule, no statistically significant differences in grip strength measurements are present between patients with and without PA (Table 4).

### PROs

The median scores of the MHQ subscales: general functioning (65.0 compared to 75.0, *p* = 0.018), esthetics (93.8 compared to 100.0, *p* = 0.037) and satisfaction (75.0 compared to 91.7, *p* = 0.042) were statistically significant poorer in the patients with PA (Table 6). Also, the median total MHQ score was statistically significant poorer in patients with PA (83.0 compared to 90.5, *p* = 0.044) (Table 4). Regarding the SF-36, physical functioning (median 95.0 versus 100.0, *p* = 0.028) was statistically significant poorer in patients with PA.

**Table 6:** PROs: differences between patients with and without PA

PROs	PA (*N* = 23) Median (IQR)	No PA (*N* = 50) Median (IQR)	Difference in medians	*p* value
DASH	6.7 (2.5; 24.2)	3.3 (0.6; 11.8)	3.4	0.094
PRWE				
Pain	8.0 (0.0; 15.0)	5.5 (0.0; 14.5)	2.5	0.661
Function	10.0 (2.0; 18.0)	4.5 (0.0; 12.0)	5.5	0.187
Total	13.0 (3.5; 21.0)	8.3 (1.4; 20.1)	4.7	0.424
MHQ				
General function	65.0 (50.0; 80.0)	75.0 (65.0; 95.0)	10.0	0.018*
Activities general life	95.0 (80.0; 100.0)	100.0 (85.0; 100.0)	5.0	0.189
Work	100.0 (80.0; 100.0)	95.0 (88.8; 100.0)	5.0	0.789
Pain	85.0 (75.0; 100.0)	90.0 (80.0; 100.0)	5.0	0.248
Esthetics	93.8 (68.8; 100.0)	100.0 (93.8; 100.0)	6.4	0.037*
Satisfaction	75.0 (41.7; 95.8)	91.7 (70.8; 100.0)	16.7	0.042*
Total	83.0 (67.1; 91.0)	90.5 (81.0; 95.5)	7.5	0.044*
SF-36				
Physical functioning	95.0 (75.0; 95.0)	100.0 (90.0; 100.0)	5.0	0.028*
Social functioning	100.0 (87.5; 100.0)	100.0 (87.5; 100.0)	0.0	0.889
Rolemodel physical problem	100.0 (50.0; 100.0)	100.0 (100.0; 100.0)	0.0	0.226
Rolemodel emotional problem	100.0 (100.0; 100.0)	100.0 (100.0; 100.0)	0.0	0.474
Mental health	88.0 (80.0; 92.0)	84.0 (75.0; 92.0)	4.0	0.469
Vitality	75.0 (60.0; 85.0)	70.4 (60.0; 85.0)	4.6	0.650
Pain	89.8 (67.3; 100.0)	79.6 (67.3; 100.0)	10.2	0.942
General health experience	80.0 (65.0; 85.0)	72.5 (63.8; 85.0)	7.5	0.277
Health change	50.0 (50.0; 50.0)	50.0 (50.0; 63.8)	0.0	0.442
Physical component	88.7 (69.3; 95.0)	88.4 (81.0; 92.5)	0.3	0.972
Mental component	87.6 (83.4; 94.3)	89.2 (79.1; 93.1)	1.6	0.669

Results of Mann–Whitney *U* test

*N* number of patients, *IQR* interquartile range, *PA* posttraumatic arthritis, *VAS* Visual Analogue Scale, *DASH* disability of arm, shoulder and hand questionnaire, *PRWE* patient rated wrist evaluation, *MHQ* Michigan Hand Questionnaire, *SF-36* Short Form (36) Health Survey

*Statistical significance

#### Regression analysis

Flexion/extension arc of motion was a statistically significant explanatory variable for DASH, PRWE function and PRWE total. Although not statistically significant, flexion/extension arc of motion did seem to be an important explanatory variable for the PRWE pain and MHQ total (Table 7). Percentage of grip strength was an explanatory variable in the linear regression models of the total MHQ score. Type of treatment and AO/OTA fracture type were not statistically significant explanatory variables for all PROs. Regarding the regression analysis of the SF36 physical component score, all variables were removed, because no association with *p* values < 0.200 were present (Table 7).

**Table 7:** Linear regression analyses regarding PROs and explanatory variables

Dependent	Explanatory variables	Regression coefficient (SE)	*p* value of variable
DASH	Gender	− 4.5 (2.8)	0.112
	Follow-up time	− 0.2 (0.1)	0.033
	Flexion/extension arc	− 0.2 (0.1)	0.017
PRWE pain	Follow-up time	− 0.1 (0.1)	0.076
	Flexion/extension arc	− 0.1 (0.1)	0.103
PRWE function	Gender	− 8.3 (3.5)	0.023
	Follow-up time	− 0.2 (0.1)	0.090
	Flexion/extension arc	− 0.2 (0.1)	0.013
PRWE total	Gender	− 5.8 (4.1)	0.164
	Follow-up time	− 0.2 (0.1)	0.058
	Flexion/extension arc	− 0.3 (0.1)	0.029
MHQ total	Flexion/extension arc	0.2 (0.1)	0.118
	% Grip strength	0.3 (0.1)	0.027
SF 36 physical component	–	–	–
SF 36 mental component	Treatment	9.7 (3.7)	0.011

*PRO* patient rated outcome measure, *MHQ* Michigan Hand Outcomes Questionnaire, *SF-36* Short Form 36 questionnaire, *SE* standard error, *% grip strength* percentage grip strength of the affected compared to the non affected wrist

### Work

Seven patients (10%) changed or left their occupation, all because of the DRF. Four of them had signs of PA. All of them changed to less demanding work or became unemployed. Change of occupation was more prevalent in patients in physically demanding jobs pre-injury; 3 of the 6 patients with heavy occupation (50%), 2 of 18 patients with medium occupation (11%), 1 of 15 with light occupation (7%) and 1 of 29 patients with sedentary occupation (3%) changed.

## Discussion

A high prevalence of PA following a DRF in young non-osteoporotic patients was found (32%). Patients with PA had statistically significant longer radial length than patients without PA. Within the group of patients with PA, radial length was also longer in comparison to the uninjured wrist. Patients healed with a residual gap more often had PA. PA was associated with diminished flexion/ extension arc of motion and ulnar/radial deviation arc of motion. Patients healed with a residual gap more often had PA. When corrected for dominance, no statistically significant differences in grip strength measurements between patients with and without PA are present. In patients with PA the subscales ‘general functioning’, ‘esthetics’ and ‘satisfaction’ from the MHQ questionnaire were statistically significant poorer, as was the total MHQ score and the physical functioning scale of the SF-36. The DASH, PRWE function and PRWE total were statistically significant impacted by flexion/extension arc of motion.

### Prevalence of PA

The high prevalence of PA of 32% after a median follow-up of 5 years in this young population was surprising. Forward et al. presented a prevalence of 43% after a mean follow up of 38 years in non-osteoporotic patients at time of the injury [4]. The prevalence in our study might be overestimated due to the low response rate, as individuals with complaints might be more interested in participating in research activities. This assumption is supported by the fact that participants had sustained more intra-articular DRFs than non-participants. Further research on the prevalence of PA after DRFs in young patients is needed.

### Radiological measurements

DRFs in non-osteoporotic patients mainly result from high-energy trauma and therefore frequently lead to intra-articular fractures [47]. Our results are supported by literature, suggesting that DRFs that healed with a residual gap and/or overall intra-articular incongruence of ≥ 2 mm are associated with early radiographic signs of PA [3, 4, 18, 48, 49]. In addition, a systematic review recently published by our research group established that other radiological predicting factors for PA such as radial length, radial inclination, dorsal angulation and ulnar variance are presented in literature with conflicting results [3]. Regarding radiological measurements, only radial length was 1.1 mm longer at follow up in patients with PA in comparison to patients without PA. All studies reporting on the influence of radial length, reported on shortening of radial length. Most studies reported no statistically significant association with shortened radial length and the development of PA [1, 5, 50], except for Forward et al. [4]. The development of PA has multifactorial causes, such as increased stress on the articular surface that damages cells and matrices of articular cartilage and subchondral bone [2]. Overcorrection of the radial length can cause higher axial loading on the articular surface of the distal radius and therefore may contribute to the development of PA [51, 52]. In previous literature normal ranges for radiological factors have been described; ulnar variance − 4 to 2 mm [31, 32], radial length 8–17 mm [33], radial inclination 16°–29° [31, 34], dorsal angulation 0-palmar 22° [35, 36]. All measurements in this study were within these normal ranges. Although radial length seems to influence the development of PA, more research regarding these radiological predicting factors for PA is mandatory to provide constructive conclusions. Common held beliefs are dictated by the findings stated earlier that anatomical reduction of articular surfaces and absolute stable internal fixation should be pursued. In this study, no statistically significant difference regarding presence of PA between patients who were treated conservatively and operatively was found. This could suggest that anatomical reduction was achieved when patients were treated surgically and little residual incongruence was present following treatment. The relatively short follow-up period (5 years) in our study should preferably be extended in further research to get insight in the ‘natural’ course of PA in young patients with DRF. With regard to CROs, it has been reported that presence of PA did not influence aROM and grip strength after 15 year follow up [53]. However, the impact of PA after long term follow up on participation in societal roles and in the personal lives of these young people, captured with PROs remains unclear.

### CROs

Patients with PA showed a diminished flexion/extension arc and ulnar/radial deviation arc of motion. It is known that residual articular incongruence affects aROM already after a follow up of 1 year [54]. Other authors have shown that patients can maintain a high level of functioning with PA [53]. Articular incongruency is the logical result from intra-articular fractures, however in our study no statistically significant association between fracture severity as depicted by the AO/OTA classification and active range of motion was found. In addition, associated intercarpal ligamentous injuries are known to influence active range of motion following DRFs and could be an explanation for the diminished active range of motion found in our study [12, 55].

Grip strength and key pinch strength of the injured side compared to the uninjured side seemed to be affected by PA. However, when correcting for dominance with the 10% rule, this statistically significant difference resides [37]. This finding supports literature emphasizing that grip strength is not a determinant of wrist function alone, but merely a reflection of overall muscle strength and condition of a chain of muscles in the upper limb [56]. We do believe that measurement of differences in grip strength between injured and uninjured side are relevant when determining follow-up outcomes of patients who sustained a DRF. Minimal detectable change (MDC) is defined as the smallest amount of change between two measurements that indicates a real change in measurement and not being a change due to measurement error [57]. This is a statistical measurement and does not take into account change as experienced by patients. Minimal clinically important difference (MCID) is the smallest change in a measurement that a patient would notice [57]. For grip strength MDC has been reported to be 6.5 kg and MCID 19.5% or 6.5 kg in patients 1 year following surgery for DRFs [58]. This suggests that the grip strength measurements between patients with and without PA presented in our study after a median follow up duration of 5.2 years are not noticeable for patients and therefore are possibly not clinically relevant. However, since PA is a chronic, progressive disorder, grip strength differences may become clinically relevant after a longer follow-up time. Further research is needed to provide more insight in this issue.

### PROs

Pain did not differ statistically significant between patients with or without PA suggesting that pain may not be the main problem non-osteoporotic patients are facing following a DRF. In patients suffering from hand osteoarthritis pain intensity does not correlate strongly with radiographic classification [59]. The fact that the level of pain was similar in both groups might be explained by the relatively short follow-up period. However, the follow up duration was long enough to show a significantly decreased active range of motion in patients with PA compared to patients without PA. This suggests that evaluation of young patients with DRFs should not only be guided by pain but also by other domains.

Another interesting finding of the application of the different PROs was that performance of activities in daily life and work, as measured by the DASH and PRWE, questionnaires specifically designed for upper extremity functioning, was similar in patients with PA compared to those without PA. In contrast, general functioning, esthetics and satisfaction as measured by the MHQ subscales were statistically significant lower in patients with PA, as was the subscale physical functioning in the SF-36 and the total MHQ score. For future research these findings imply that other dimensions, different from those measured by the commonly used PRWE and DASH should be evaluated when measuring consequences of PA in patients with DRFs. MDC and MCID have been described to be respectively 7.7 and 17.3 for PRWE and 9.3 and 13.8 points for DASH [60]. For the MHQ none of the domains were reported to be discriminative after 3 years following volar plate fixation for DRFs, but for patients with carpal tunnel syndrome, MCIDs of 23, 13 and 8 were identified for the pain, function and work domains, respectively [61]. This suggests that the difference reported in this study in general function domain between patients with and without PA (10.0, *p* = 0.018) might not be relevant for patients. However, the study by Shauver et al. described 12 points difference on the satisfaction domain to differentiate between satisfied and unsatisfied patients. Therefore, the difference between patients with and without PA regarding the satisfaction domain (16.7, *p* = 0.044) might be clinically relevant. Unfortunately, no MDC or MCID have been described for the domains of the SF-36. It is surprising that the more subjective domains such as esthetics and satisfaction were statistically significant associated with PA and not the domains reporting on pain and (daily) functioning. The impact of PA for patients following DRFs in everyday life, while there is limited aROM, does not seem to be significant. However patients with PA are less satisfied. Further research should clarify the specific reasons for dissatisfaction.

Waljee et al. recently described a core set of domains that should be reported in order to get insight into outcomes after a DRF for clinical or research purposes: performance, PROs, pain, complications and radiographs [62]. Domains found in our study to be statistically significant different between participants with and without PA such as satisfaction and esthetics, are however lacking in this core set. To report about patient satisfaction is becoming increasingly important, since in modern medicine patient-centered health care is emphasized. Decision making in healthcare has shifted from a paternalistic model to informed decision-making and shared decision-making. There is evidence suggesting that shared decision-making does facilitate positive health outcomes and improves satisfaction [63]. In the future, it might be beneficial to further explore which elements of Waljee’s proposed core set are relevant in the clinical follow up of non-osteoporotic patients following DRFs [62, 64]. With regard to the PROs, we recommend the use of the MHQ subscales in clinical practice and post-injury DRF research, as this instrument seems to distinguish between patients with or without PA in the univariate analyses.

In literature, the few studies reporting on associations between CROs and PROs following DRFs describe these results at short-term follow up [65–68]. Chung et al. use two questions of the MHQ subscale satisfaction regarding range of motion and grip strength to determine cut-off points for satisfaction 3 month following a DRF. Optimal cut-points to distinguish satisfaction from dissatisfaction were met when patients recovered 65% of their grip strength and 95% of the wrist arc of motion [65]. Shauver et al. describe linear regression analyses revealing that 3 months following a DRF, patient’s education, income, age at time of surgery and all measured outcome variables (grip strength difference, pinch strength difference, flexion, extension, active arc of motion, ulnar deviation, radial deviation, pronation, supination) accounted for 37% of the explained variance in total MHQ score [66]. Souer et al. describe a model where independent predictors pain (*F* = 61.16, *p* < 0.001) and forearm rotation (*F* = 27.39, *p* < 0.001) account for 71% of the explained variance of the DASH at a median follow-up of 6 months [68]. Our results support the finding that especially active range of motion is an important determinant of PROs. Type of treatment and AO/OTA fracture type did not seem to influence PROs. In addition, the influence of predicting factors seems to become less prominent with a longer follow-up duration as the explained variance in our study was lower than the earlier mentioned studies [66, 68]. Future research should be aimed at determining a complete overview of factors influencing PROs following DRFs in non-osteoporotic patients at early, but also at longer follow-up period. From our study, we conclude that the development of PA impacts active range of motion. Patients perceive diminished general functioning and satisfaction following PA and diminished active range of motion. This insight could direct rehabilitation strategies and can be used to counsel these patients on expected outcome.

Our results reporting 10% change of occupation following a DRF are likely to be of major interest for patients. No statistically significant association with PA was found. Although all patients reported to have changed occupation because of the injury, this percentage might be a normal change of occupation in this population. It is striking however that all patients changed to a physically less demanding occupation and change of occupation occurred more often in physically demanding jobs. This suggests that patients more often need to adapt their working environment following a DRF when having a physically demanding occupation.

### Strengths and weaknesses

Where most studies report on osteoporotic patients, sometimes combined with non-osteoporotic patients, we report on a young non-osteoporotic population who sustained a DRF 4–11 years ago. As such, we contribute to the knowledge PA and its association with radiological measurements, CROs and PROs in young patients. In addition, we have used measurements of the uninjured wrist as control when calculating the percentage of grip strength. Large variations between patients are accounted for in this way. The active range of motion and grip strength measurements were performed by one hand therapist for consistency. Measurements were performed in a fixed sequence. As a consequence however, fatigue effects may have influenced our results. In future research, a random sequence of measurements should be considered. Intraobserver and interobserver variability of radiological measurements and AO/OTA fracture classifications of DRFs on radiographs is known to be moderate [29, 69]. To eliminate interobserver variability, all measurements on radiographs were performed by one specialized radiologist. It has to be acknowledged that, although all radiographs have been performed according to protocol, measurement accuracy can be influenced by the quality of the radiograph taken and computed tomography could be more sensitive. The patients in our study did not have radiographic measurements out of normal ranges as described in literature [31–36]. Still, a prevalence of 32% PA at a relatively short follow up duration of 5 years is a substantial portion and is likely to progress with longer follow up duration. Our response rate was low, presumably because this population is young and has moved for study or work purposes and therefore many current addresses could not be retrieved. The included number of 73 patients might be insufficient to draw firm conclusions. However, in most studies describing populations after DRF the number of patients included in this study is not exceeded [10, 65, 70, 71]. Moreover those studies do not report response rates [4, 18, 20, 48]. Our results contribute to the knowledge on how to improve outcome and diminish PA in the future. However, studies with longer follow up duration are mandatory to gain more insight in the influence of progressed PA on outcome.

## Conclusion

Non-osteoporotic patients had a considerably high prevalence of PA following DRFs, despite relatively short follow-up time. Correction of radial length should be performed as precise as possible, as overcorrection may induce PA. PA is associated with diminished flexion/extension and ulnar/radial deviation, irrespective of AO/OTA fracture type. Grip strength seems to be merely a determinant of strength and condition of the complete upper arm, as radiological measurements and PA does not seem to influence it. Non-osteoporotic patients following DRFs perceived diminished general functioning and dissatisfaction, which was impacted by the diminished active range of motion. Pain or impaired general health status were not reported. The PRO MHQ might be a valuable evaluation tool in this patient group. Change of occupation following DRFs should receive attention in further research.
